# Role of Trusted Sources and Behavioral Beliefs in Promoting Mitigation Behaviors During the COVID-19 Pandemic: Survey Study

**DOI:** 10.2196/37454

**Published:** 2022-07-13

**Authors:** Bridget L Hanson, Kari Finley, Jay Otto, Nicholas J Ward

**Affiliations:** 1 Center for Health and Safety Culture Montana State University Bozeman, MT United States

**Keywords:** behavioral beliefs, health literacy, vaccination, trusted sources, social media, vaccine hesitancy, health information, masking, healthcare, public health, health beliefs

## Abstract

**Background:**

During the ongoing COVID-19 pandemic and in preparation for future public health crises, it is important to understand the relationship between individuals’ health beliefs, including their trust in various sources of health information, and their engagement in mitigation behaviors.

**Objective:**

We sought to identify relationships between trust in various sources of health information and the behavioral beliefs related to vaccination and mask wearing as well as to understand how behavioral beliefs related to vaccination differ by willingness to be vaccinated.

**Methods:**

We conducted an online survey of 1034 adults in the United States and assessed their trust in federal, local, and media sources of health information; their beliefs about vaccination; and their masking intention and vaccination willingness.

**Results:**

Using regression, masking intention was predicted by trust in the World Health Organization (*P*<.05) and participants’ state public health offices (*P*<.05), while vaccine willingness was predicted by trust in participants’ own health care providers (*P*<.05) and pharmaceutical companies (*P*<.001). Compared to individuals with low willingness to be vaccinated, individuals with high willingness indicated greater endorsement of beliefs that vaccines would support a return to normalcy, are safe, and are a social responsibility (*P*<.001 for all).

**Conclusions:**

Results can be used to inform ongoing public health messaging campaigns to manage the COVID-19 pandemic and increase readiness for the next pandemic. Additionally, results support the need to bolster the public’s trust in health care agencies as well as to enhance trust and respect in health care providers to increase people’s adoption of mitigation behaviors.

## Introduction

COVID-19, the illness caused by the novel SARS-CoV-2 virus, has caused a global health crisis. As of early 2022, more than 78 million cases and 930,000 COVID-19 deaths have been reported in the United States [1]. Individual engagement in mitigation behaviors like mask wearing and vaccination is critical for decreasing transmission of the virus. However, despite clear evidence of the effectiveness of both masking and vaccines and the widespread availability of both, participation in these mitigation behaviors is inconsistent in the United States [1,2].

In many models and explanatory theories of health behavior, especially planned behaviors like mask wearing and vaccination, beliefs are predictors of behaviors [3]. During the COVID-19 pandemic, beliefs have been affected by limited and changing information due to the novelty of the virus as well as misinformation spread both deliberately and unintentionally [4-6].

The spread of misinformation has compounded an already eroding trust in government agencies, including public health agencies and organizations [7,8]. Despite diminished trust in public health and polarized attitudes toward health care workers during the pandemic [9], most Americans report sustained trust in health care systems and their health care providers [10]. Availability of information from trusted sources is crucial for establishing beliefs and promoting people’s acceptance of and engagement in mitigation strategies.

The goal of this research was to identify relationships between trust in various sources of health information and the behavioral beliefs related to vaccination and mask wearing as well as to understand how behavioral beliefs related to vaccination differ by individuals’ willingness to be vaccinated. Understanding these relationships between beliefs and health behaviors that mitigate the risk and spread of COVID-19 (specifically mask wearing and vaccination) is critical for promoting uptake of mitigation behaviors among individuals who are resistant and for managing this and future pandemics. Findings can also be used to inform important lessons that can be applied to other current public health issues and better prepare health care workers, public health officials, and others to respond to future crises.

## Methods

### Survey

We administered an online survey in October 2020 to a convenience sample of adults in the United States using a Qualtrics purchased panel (Qualtrics International Inc) [11,12]. We developed the survey based on the reasoned action approach to health behaviors [3] and informed by two small pilot tests (total n=210). The final survey included a variety of questions to assess beliefs, attitudes, and behaviors related to COVID-19. Of interest in this paper are questions about behavioral intention and willingness, trust in sources of information, beliefs associated with vaccination, and demographics. See Multimedia Appendix 1 for a copy of study items from the survey.

Mask wearing intention and vaccine willingness were each assessed with a single question. Participants’ trust in various sources of information was assessed by asking “How much do you trust information from the following sources about COVID-19?” and participants rated each source separately. Participants’ beliefs associated with vaccination were assessed through 7 items exploring safety, concern about side effects, perception of social responsibility, and similar beliefs. All survey questions used 7-point scales; higher scores indicated greater behavioral intention/willingness, trustworthiness, and agreement.

Demographic information included sex, age, race, income, geography (urban, suburban, rural), and state of residence.

### Data Analysis

Data analyses were multiple regressions and multiple analysis of variance (MANOVA), which were conducted in SPSS (version 27; IBM Corp), with α set at .05. Missing data were minimal (<2% for each item), missing at random, and excluded from analyses with pairwise deletion.

### Ethical Considerations

The study was reviewed and determined exempt by the Montana State University Institutional Review Board (FWA: 00000165; protocol #KF100720). Participants provided informed consent before completing the survey.

## Results

The sample consisted of 1034 adults residing in the United States. A description of the sample is provided in Multimedia Appendix 2. Descriptive statistics of the study variables are shown in Table 1.

To understand the relationship between trusted sources and COVID-19 mitigation behaviors, we conducted two multiple linear regression models that predicted (1) intention to wear a mask and (2) willingness to be vaccinated (as the dependent variables) based on reported trust. These regressions included the 10 variables assessing trust in various sources of information about the COVID-19 pandemic and demographic variables of age, sex (0=male, 1=female), education, income, and geography (1=rural, 2=suburban, 3=urban) as predictors using the enter method. Regarding potential multicollinearity, we noted that while some predictor variables were correlated (with the highest correlation between trust in the Centers for Disease Control and Prevention and trust in the World Health Organization, *r*=.79), the variance inflation factor did not exceed 3.5 for any predictor in either model. Therefore, we retained all predictor variables in both models [13]. Both regression models were significant overall and significant predictors differed between the models (Table 2).

The model for participants’ intention to wear a mask was significant (*F*_15,923_=13.32; *P*<.001; *R*^2^=.18; *f*^2^=.22). Three trusted sources were significant predictors. Trust in the World Health Organization and trust in the state’s public health office were both positively associated with intention to wear a mask, while trust in the White House/President was negatively associated and was the strongest predictor. Demographic variables of age and sex were significant predictors, with increasing age associated with greater intention to mask and women (more than men) intending to mask.

The model for willingness to be vaccinated was also significant (*F*_15,916_=18.73; *P*<.001; *R*^2^=.23; *f*^2^=.30). In this model, participants’ trust in their local health care provider and trust in pharmaceutical/drug companies were significant predictors and both positively associated with willingness to be vaccinated. The only demographic variable that predicted vaccination willingness was geography, with willingness to be vaccinated increasing as geographic density increased (ie, urban participants were more willing to be vaccinated than rural or suburban participants).

**Figure 1:**
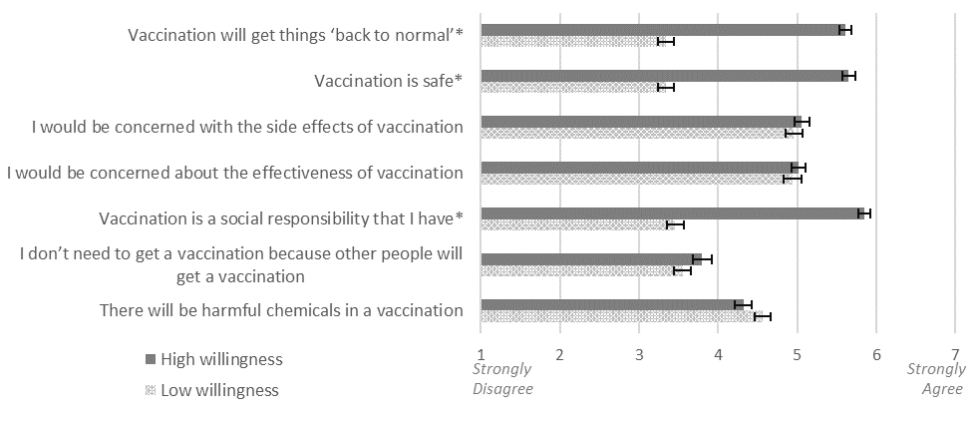
Vaccine-related beliefs by willingness to be vaccinated. Error bars represent standard errors. Table 1 provides the complete wording of each item. *P<.001.

To better understand differences in beliefs between those willing to be vaccinated and those unwilling to be vaccinated, we grouped participants based on their willingness response into low (responses of 1 or 2; n=299) and high (responses of 6 or 7; n=356) and conducted a MANOVA with the 7 beliefs about vaccination as the dependent variables. The overall MANOVA was significant (*F*_7, 647_=70.42; *P*<.001; partial *η*^2^=.43). Applying a Bonferroni correction for multiple comparisons adjusted the α for follow-up analysis of variance (ANOVA) to .007. The ANOVA revealed significant differences between groups for 3 of the 7 beliefs. Compared to those with low willingness to be vaccinated, participants with high willingness agreed significantly more that vaccination will get things back to normal (*F*_1, 653_=306.38; *P*<.001; partial *η*^2^=.32), is safe (*F*_1, 653_=364.55; *P*<.001; partial *η*^2^=.36), and is a social responsibility (*F*_1, 653_=338.56; *P*<.001; partial *η*^2^=.34) (Figure 1).

**Table 1:** Study variable descriptives.

		Participant answers, n	Mean (SD)
**Behavioral intention/willingness**			
	Intent to wear a mask	1026	5.69 (1.94)
	Willingness to be vaccinated	1020	4.18 (2.24)
**Trusted sources**			
	Trust World Health Organization	1030	4.64 (2.07)
	Trust Centers for Disease Control and Prevention	1027	4.92 (1.85)
	Trust White House/President	1029	3.70 (2.28)
	Trust state’s public health office	1025	4.76 (1.84)
	Trust local public health office	1026	4.82 (1.77)
	Trust health care provider	1027	5.25 (1.75)
	Trust pharmaceutical/drug companies	1027	4.53 (1.84)
	Trust television news stations	1022	4.15 (1.92)
	Trust social media	1023	3.64 (2.05)
	Trust work colleagues/classmates	1024	4.22 (1.84)
**Beliefs related to vaccination**			
	“Getting an FDA-approved^a^ vaccination to prevent COVID-19 will get things ‘back to normal.’”	1023	4.46 (1.86)
	“Getting an FDA-approved vaccination to prevent COVID-19 is safe.”	1020	4.51 (1.76)
	“I would be concerned with the side effects of an FDA-approved vaccination to prevent COVID-19.”	1018	4.86 (1.74)
	“I would be concerned about the effectiveness of an FDA-approved vaccination to prevent COVID-19.”	1017	4.83 (1.73)
	“Getting an FDA-approved vaccination to prevent COVID-19 when it becomes available is a social responsibility that I have.”	1020	4.72 (1.86)
	“I don’t need to get an FDA-approved vaccination to prevent COVID-19 because other people will get a vaccination.”	1018	3.59 (2.00)
	“There will be harmful chemicals in an FDA-approved vaccination to prevent COVID-19.”	1019	4.30 (1.83)

^a^FDA: Food and Drug Administration.

**Table 2:** Regression models to predict mask wearing intention and vaccine willingness.

Predictor	Intent to mask			Willingness to be vaccinated	
	*B* (95% CI)	β	*B* (95% CI)		β
Trust World Health Organization	.13 (.03 to .22)	.13^a^	.06 (–.05 to .17)		.06
Trust Centers for Disease Control and Prevention	.10 (–.02 to .21)	.09	.08 (–.05 to .21)		.06
Trust White House/President	–.15 (–.21 to –.09)	–.18^b^	.06 (–.01 to .13)		.06
Trust state’s public health office	.12 (.01 to .23)	.11^a^	.03 (–.09 to .16)		.03
Trust local public health office	–.03 (–.15 to .09)	–.03	.06 (–.07 to .19)		.05
Trust health care provider	.09 (–.01 to .19)	.08	.14 (.03 to .25)		.11^a^
Trust pharmaceutical/drug companies	.03 (–.07 to .12)	.02	.19 (.09 to .30)		.16^b^
Trust television news stations	.01 (–.09 to .10)	.01	.02 (–.08 to .13)		.02
Trust social media	–.03 (–.12 to .06)	–.03	.01 (–.09 to .12)		.01
Trust work colleagues/classmates	–.04 (–.13 to .06)	–.04	.03 (–.07 to .14)		.03
Age	.01 (.01 to .02)	.11^a^	.00 (–.01 to .01)		.00
Sex	.53 (.28 to .79)	.14^b^	–.19 (–.48 to .01)		–.04
Education	–.01 (–.09 to .07)	–.01	.06 (–.03 to .15)		.05
Income	.06 (–.03 to .14)	.05	.01 (–.09 to .10)		.01
Geography	–.05 (–.21 to .12)	–.02	.27 (.08 to .45)		.09^a^

^a^*P*<.05.

^b^*P*<.001.

## Discussion

This study identified the relationship between trusted sources of information regarding COVID-19 and individuals’ intention to wear a mask and willingness to get vaccinated and provides useful information for promoting public health during the current COVID-19 pandemic as well as for increasing capacity to respond efficiently and effectively in the future.

The spread of health misinformation has risen to the level of an “urgent threat,” according to the US Surgeon General, and combating misinformation is a priority focus of his office [14]. Identifying trusted sources is a critical first step in spreading accurate messaging to the public and communicating public health science to combat misinformation [15]. In our study, trust in the World Health Organization and state public health offices was positively associated with intention to wear a mask, suggesting that information from these sources should be amplified and that bolstering the public’s trust in these offices could support individuals’ masking behaviors. Trust in the White House was negatively associated with masking intention, which is unsurprising given our survey was conducted in October 2020, and the Trump administration did not consistently promote or encourage masking [16].

Different predictors were associated with participants’ willingness to be vaccinated. Trust in their personal health care provider and the pharmaceutical industry predicted willingness to be vaccinated. Ensuring trust in health care providers and promoting them as health information sources are necessary for the public to seek and obtain accurate health information [17]. Additionally, low trust in pharmaceutical companies could be hampering vaccination [18-21].

Since the survey was conducted before vaccines were approved in the United States, we lack data on actual vaccine behavior, which is an important limitation. Nonetheless, willingness is an important predictor of behavior and, given the lagging uptake of vaccination, promoting trusted sources continues to be important. For all mitigation behaviors, including masking and vaccination, understanding who the intended audience considers to be a trusted source for health information is an important consideration in efforts to provide public health information. Effective health interventions should be tailored to the intended audience, including using trusted sources to deliver the information [22].

Further, our research found that, compared to those with low willingness to be vaccinated, participants with high willingness indicated greater endorsement of beliefs that vaccination will get things back to normal, is safe, and is a social responsibility. This represents an important opportunity to frame communication about vaccination in ways that promote these protective beliefs, such as fostering a sense of social responsibility through communication that seeks to cultivate a sense of community and intentionally promotes a shared vision. Efforts may also seek to promote health literacy, as health literacy includes understanding the importance of protecting ourselves as well as others [23].

Interestingly, while beliefs about social responsibility did differ based on willingness, belief that others getting vaccinated negates one’s own need for vaccination did not differ. Beliefs about vaccine effectiveness, side effects, or chemicals also did not differ based on willingness, suggesting that messaging around these topics may be less effective in promoting vaccination behaviors.

The data were gathered from a convenience sample of adult participants and therefore may not generalize to all people or communities in the United States. Additionally, behavioral intention and willingness were measured with single survey items, thereby preventing reliability estimates. Future research might explore behavioral beliefs related to mitigation behaviors as well as the mitigation behaviors directly with additional samples and using alternative instruments.

Despite limitations, the results have actionable implications. Taken together, findings from this study can be used to inform communication efforts that empower people to find accurate information regarding their health decisions, including engagement in mitigation efforts during the COVID-19 pandemic. Lessons can also be applied to the development of relevant messages targeting specific beliefs and encouraging behaviors that promote public health more quickly and effectively during the next pandemic or another public health crisis.

## Appendix

Multimedia Appendix 1 Survey items.

Multimedia Appendix 2 Sample description.

## Abbreviations

ANOVA: analysis of variance
MANOVA: multiple analysis of variance
