# Supplementary material for: Role of Trusted Sources and Behavioral Beliefs in Promoting Mitigation Behaviors During the COVID-19 Pandemic: Survey Study
Source: JMIR Hum Factors. 2022 Jul 13;9(3):e37454. doi: 10.2196/37454 (PMC9285667; doi:10.2196/37454)
Supplement: Multimedia Appendix 1 [file humanfactors_v9i3e37454_app1.docx]

**Survey Items**

**Mask wearing intention:**

I intend to always wear a mask when around people (within 6 feet) who don’t live in my household until the end of the COVID-19 pandemic.

*Response options: strongly disagree (1), moderately disagree (2), somewhat disagree (3), neither agree nor disagree (4), somewhat agree (5), moderately agree (6), strongly agree (7)*

**Vaccine willingness:**

How willing are you to be vaccinated today if a free and FDA-approved vaccination to prevent COVID-19 was available?

*Response options: not at all willing (1), (2), (3), moderately willing (4), (5), (6), extremely willing (7)*

**Trusted sources:**

How much do you trust information from the following sources about COVID-19?

World Health Organization (WHO)

Centers for Disease Control and Prevention (CDC)

The White House/President

Your State’s Public Health Office

Your Local (County or City) Public Health Office

Your Local Healthcare Provider (i.e. your doctor)

Pharmaceutical/Drug Companies

Television News Stations (i.e. PBS, CBS, NBC, FOX, etc.)

Social Media (i.e. posts on Facebook, Twitter feeds)

Your Work Colleagues/Classmates

*Response options: not at all trustworthy (1), (2), (3), moderately trustworthy (4), (5), (6), extremely trustworthy (7)*

**Beliefs about vaccination:**

Getting an FDA-approved vaccination to prevent COVID-19 will get things “back to normal.”

Getting an FDA-approved vaccination to prevent COVID-19 is safe.

I would be concerned with the side effects of an FDA-approved vaccination to prevent COVID-19.

I would be concerned about the effectiveness of an FDA-approved vaccination to prevent COVID-19.

Getting an FDA-approved vaccination to prevent COVID-19 is a social responsibility that I have.

I don’t need to get an FDA-approved vaccination to prevent COVID-19 because other people will get a vaccination.

There will be harmful chemicals in an FDA-approved vaccination to prevent COVID-19.

*Response options: strongly disagree (1), moderately disagree (2), somewhat disagree (3), neither agree nor disagree (4), somewhat agree (5), moderately agree (6), strongly agree (7)*

**Demographics:**

In what state do you currently reside?

*Dropdown with options of U.S. states, D.C., territories, and “I do not reside in the United States”*

What best describes where you live?

*Response options: Urban (50,000 or more people) (1), Suburban (2,500 to 50,000 people) (2), Rural (2,500 people or less) (3)*

What is the highest level of education that you completed or the highest degree you have received?

*Response options: Less than high school degree (1) , high school graduate (high school diploma or equivalent including GED) (2), some college but no degree (3), Associate degree in college (2year) (4), Bachelor’s degree in college (4-year) (5), master’s degree (6), doctoral degree (7), professional degree (JD, MD) (8)*

How old are you?

How would you describe yourself?

*Response options: Male (1), Female (2), Transgender (3), Non-binary gender (4), Other (5), I prefer not to answer (99)*

Are you Spanish, Hispanic, or Latino or none of these?

*Response options: Yes (1), None of these (2)*

Choose one or more races that you consider yourself to be:

*Response options: White (1), Black or African American (2), American Indian or Alaska Native (3), Asian (4), Native Hawaiian or Pacific Islander (5), Other (6), I prefer not to answer (99)*

Is your annual household income from all sources –

*Response options: Less than $25,000 (1)*, *$25,000 to less than $35,000 (2)*, *$35,000 to less than $50,000 (3)*, *$50,000 to less than $75,000 (4)*, *$75,000 or more (5)*, *I prefer not to answer (99)*
