# Supplementary material for: Role of Trusted Sources and Behavioral Beliefs in Promoting Mitigation Behaviors During the COVID-19 Pandemic: Survey Study
Source: JMIR Hum Factors. 2022 Jul 13;9(3):e37454. doi: 10.2196/37454 (PMC9285667; doi:10.2196/37454)
Supplement: Multimedia Appendix 2 [file humanfactors_v9i3e37454_app2.docx]

**Sample**

The sample consisted of 1,034 adults (age 18 or older); participants were from all 50 states and the District of Columbia. When compared to 2019 U.S. Census estimates, the distribution of participants approximated the population distribution within 1.2% for each state with three exceptions. Michigan was undersampled by 1.7%, and Georgia and New York were oversampled by 1.6% and 7.1%, respectively.

About half (*n*=524; 50.7%) of the respondents were female, and ages ranged from 18 to 70 (*M =* 44.9 years, *SD* = 16.6 years). Most (*n*=662; 64.0%) respondents identified as White, with 27.5% (*n*=284) Black, 1.9% (*n*=20) Asian, 1.7% (*n*=18) American Indian or Alaska Native, 0.5% (*n*=5) Native Hawaiian or Pacific Islander, and 4.9% (*n*=51) selected “other” or “prefer not to answer.” For ethnicity, 24.3% (*n*=251) identified as Spanish, Hispanic, or Latino. For geography, 41.3% (*n*=427) lived in an urban environment, 40.8% (*n*=422) suburban, and 17.9% (*n*=185) rural. Finally, 25.1% (*n*=260) had a high school education or less and 45.0% (*n*=465) had a Bachelor’s degree or more education; and 49.0% (*n*=507) had an annual household income of less than $50,000.
